# Supplementary material for: Content-rich biological network constructed by mining PubMed abstracts
Source: BMC Bioinformatics. 2004 Oct 8;5:147. doi: 10.1186/1471-2105-5-147 (PMC528731; doi:10.1186/1471-2105-5-147)
Supplement: Additional File 2 — The original results of the above study (non-essential files are deleted to keep the file size under the limit set by BMC bioinformatics). [file 1471-2105-5-147-S2.bz2 › chilibotAdditionalFile2/dip20/1ID9194558E1/html/BAX_BCL2L1.html]

 


 **BAX** and **BCL2L1** 
  
Found 1196 abstracts in PubMed, retrieved 20.  
 

 What does Google say? 
 PDF only 
| .edu only 

---

**Interactive relationship** (e.g. stimulation, inhibition, etc)

**Inhibitory relationship**- ... Bcl xl  [ **BCL2L1** ]  induction suppresses a p53 and  **Bax**  dependent apoptotic pathway.  Ref: 12855666 Clin Cancer Res, 2003
- It was reported that SCF c kit, Bcl 2 and Bcl xl  [ **BCL2L1** ]  inhibited the apoptosis while caspase 3, Fas,  **Bax**  and clusterine stimulated it.  Ref: 12861841 Zhonghua Nan Ke Xue, 2003
- A small inhibitor of the interaction between  **Bax**  and Bcl X  [ **BCL2L1** ]  L can synergize with methylprednisolone to induce apoptosis in Bcl X  [ **BCL2L1** ]  L overexpressing breast cancer cells.  Ref: 12884026 J Cancer Res Clin OncolJ Cancer Res Clin Oncol, 2003
- We have identified a small inhibitor of the interaction between  **Bax**  and Bcl X  [ **BCL2L1** ]  L that can synergize with methylprednisolone to induce apoptosis in Bcl X  [ **BCL2L1** ]  L overexpressing breast cancer cells.  Ref: 12884026 J Cancer Res Clin OncolJ Cancer Res Clin Oncol, 2003
**Neutral relationship**- Using an assay based on biosensor technology, we screened a chemical library of 1 00 compounds for inhibitors of the interaction between  **Bax**  and Bcl X  [ **BCL2L1** ]  L.  Ref: 12884026 J Cancer Res Clin OncolJ Cancer Res Clin Oncol, 2003
- To identify inhibitors of the interaction between  **Bax**  and Bcl X  [ **BCL2L1** ]  L.  Ref: 12884026 J Cancer Res Clin OncolJ Cancer Res Clin Oncol, 2003

**Non-interactive relationship** (e.g. studied together, co-existance, homology, etc.)

- **Bax**  was detected in homogenate and cytosol, and its level was not affected by ethanol.CONCLUSIONS DLPC, at a dose contained in PPC, reproduces the antiapoptotic actions of PPC through a reduction in cytosolic cytochrome c concentration and caspase 3 activity, possibly in association with up regulation of Bcl x  [ **BCL2L1** ]  L expression.  Ref: 12824822 Alcohol Clin Exp Res, 2003
- **Bax**  and Bcl xL  [ **BCL2L1** ]  proteins were not changed, although Bcl 2 proteins increased with troglitazone.  Ref: 12840598 Am J Nephrol< MedlineTA>Am J Nephrol, 2003
- SN38 induced p53,  **Bax** , Bcl xl  [ **BCL2L1** ] , and p53 dependent p21 WAF1 protein accumulation.  Ref: 12855666 Clin Cancer Res, 2003
- **Bax** , a death promoting protein, was upregulated and Bcl x  [ **BCL2L1** ]  L, a death inhibiting protein, was downregulated.  Ref: 12830326 Eur J Nucl Med Mol Imaging< MedlineTA>Eur J Nucl Med Mol Imaging, 2003
- The protein contents of cytochrome c, procaspase 3, caspase 3, Bcl x  [ **BCL2L1** ]  L, and  **Bax**  were analyzed by Western blot and quantified by densitometry.  Ref: 12824822 Alcohol Clin Exp Res, 2003
- The  **Bax**  Bcl xl  [ **BCL2L1** ]  ratio changed little.  Ref: 12855666 Clin Cancer Res, 2003
- Experimental Design Using wild type HCT116, p53 null,  **Bax**  null, or p21 WAF1 null isogenic derivatives, we measured expression of regulators of cellular response, and associated growth arrests or apoptosis, after SN38 treatment, with or without antisense mediated Bcl xl  [ **BCL2L1** ]  knockdown.  Ref: 12855666 Clin Cancer Res, 2003
- AIMS To correlate the expression of a series of apoptotic and oncogene markers including p53, Bcl 2,  **BAX** , Bcl XL  [ **BCL2L1** ] , p21WAF, 1 CIP1, cyclin D1, HER 2 neu in thymic epithelial tumours with histological type, stage and resectability and to determine whether the information on HER 2 neu would be valuable in identifying patients who are eligible for anti HER 2 neu treatment.  Ref: 12877732 Histopathology, 2003
- In wild type HCT116, but not in  **Bax**  null cells, Bcl xl  [ **BCL2L1** ]  knockdown induced a shift in response from drug induced senescence to apoptosis, and enhanced the global cytotoxicity of SN38.  Ref: 12855666 Clin Cancer Res, 2003
- METHODS We performed Northern blot analysis for  **bax** , bcl x  [ **BCL2L1** ]  L and bcl 2 at both temperatures.  Ref: 12824868 Eur J Endocrinol, 2003
- ... other Bcl 2 family proteins including  **Bax** , Bcl 2, Bcl XL  [ **BCL2L1** ] , and Bag remained unchanged.  Ref: 12874837 J Cell Biochem, 2003
- RESULTS Ribonuclease protection assay data indicate an age related increase in  **Bax** , Bcl xL  [ **BCL2L1** ] , and procaspase 3 messenger RNA expression in aged compared with young rats.  Ref: 12862407 Am J RhinolAm J Rhinol, 2003
- Expression of  **BAX**  and Bcl XL  [ **BCL2L1** ]  were probed in the cells treated with or without Erbitux.Results.  Ref: 12850474 J Surg Res, 2003
- There was no difference found in the expression of proapoptotic  **Bax**  and Bcl X  [ **BCL2L1** ]  s or antiapoptotic Bcl 2 and Bcl X  [ **BCL2L1** ]  L genes nor in the expression of the tumor suppressor gene p53.  Ref: 12885947 J Leukoc Biol, 2003
- No change was detected in Bcl 2,  **Bax** , or Bcl xL  [ **BCL2L1** ]  proteins.  Ref: 12830043 J Thorac Cardiovasc Surg, 2003
- Remarkably, these events occurred in the absence of any reduction in the expression of the Bcl 2 family members Bcl 2, Mcl 1 and Bcl XL  [ **BCL2L1** ] , or any change in the proapoptotic molecules Bad or  **Bax** .  Ref: 12842827 Am J Physiol Gastrointest Liver Physiol< MedlineTA>Am J Physiol Gastrointest Liver Physiol, 2003
- Immunoprecipitation studies revealed that Nbk interacts with Bcl x  [ **BCL2L1** ]  L and Bcl 2 but not with  **Bax** .  Ref: 12853473 EMBO J< MedlineTA>EMBO J, 2003
- In these conditions, microscopic analysis revealed chromatin condensation in dying cells and the Bcl 2, Bcl x  [ **BCL2L1** ]  L  **Bax**  mRNA balance was altered.  Ref: 12832039 FEBS Lett, 2003
